# Transcranial Direct Current Stimulation for Orthopedic Pain: A Systematic Review with Meta-Analysis

**DOI:** 10.3390/brainsci14010066

**Published:** 2024-01-09

**Authors:** William Adams, Sherina Idnani, Joosung Kim

**Affiliations:** 1 Department of Kinesiology and Sport Sciences, University of Miami, Coral Gables, FL 33146, USA; w.adams@miami.edu (W.A.); sxi175@miami.edu (S.I.); 2 Department of Health and Human Performance, Texas State University, San Marcos, TX 78666, USA

**Keywords:** brain stimulation, chronic pain, joint injuries, neurorehabilitation

## Abstract

(1) Background: Transcranial direct current stimulation (tDCS) appears to alleviate chronic pain via a brain-down mechanism. Although several review studies have examined the effects of tDCS on patients with chronic pain, no systematic review or meta-analysis has comprehensively analyzed the effects of tDCS on chronic orthopedic joint pain in one study. We aim to evaluate the effectiveness of tDCS for pain reduction in chronic orthopedic patients; (2) Methods: A comprehensive search of five electronic databases (Medline, Embase, Web of Science, CINAHL, and Cochrane) was performed. Only randomized controlled trials that compared tDCS with a control intervention were included. Eighteen studies met our inclusion criteria. We identified four categories of chronic orthopedic pain: knee (k = 8), lower back (k = 7), shoulder (k = 2), and orofacial pain (k = 1). Random effect models were utilized, and a sensitivity analysis was conducted in the presence of significant heterogeneity. Studies within each pain condition were further classified according to the number of treatment sessions: 1–5 sessions, 6–10 sessions, and >10 sessions.; (3) Results: Significant reductions in chronic orthopedic joint pain were observed following tDCS compared to controls for knee (*g* = 0.59, *p* = 0.005), lower back (*g* = 1.14, *p* = 0.005), and shoulder (*g* = 1.17, *p* = 0.020). Subgroup analyses showed pain reductions after 6–10 tDCS sessions for knee pain and after 1–5 and >10 sessions for lower back pain; (4) Conclusions: tDCS could be considered a potential stand-alone or supplemental therapy for chronic knee and lower back pain. The effectiveness of tDCS treatment varies depending on the number of treatment sessions. Our findings suggest the importance of implementing individualized treatment plans when considering tDCS for chronic pain conditions.

## 1. Introduction

Chronic orthopedic joint pain is one of the most significant financial burdens on the healthcare system [1]. The management of chronic orthopedic joint pain presents a considerable challenge [2]. Currently, opioid medications are among the most frequently used tools to manage chronic orthopedic joint pain; however, their results are mixed, and they come with an array of detrimental side effects such as sedation, drowsiness, and a high risk of dependency [3]. It has been suggested that the ineffectiveness of opioid medications in providing long-term relief for chronic orthopedic joint pain may be due to their failure to address the central sensitization component of the patient’s pain experience [4].

Transcranial direct current stimulation (tDCS) is a non-invasive brain stimulation technique that modulates spontaneous cortical activity [5]. Traditionally, tDCS has been utilized to modify behavior, accelerate learning, and augment task performance [6,7], but recent research [8] has explored its effectiveness in modulating pain by influencing the nervous system’s central sensitization. Central sensitization is a phenomenon in which the nervous system perceives normal sensory inputs as threatening, leading to an abnormal upregulation of nociceptors [9]. Consequently, patients may experience pain during activities that ordinarily would not cause pain [8]. tDCS has emerged as a viable pain management tool due to its portability, safety, and unique non-invasive capability to directly influence brain sensitization [10,11,12].

Up to this point, the primary target for tDCS in managing chronic orthopedic joint pain has been the primary motor cortex (M1), with and without additional intervention [13,14,15]. While the mechanistic studies are still under investigation, a number of randomized controlled trials have reported reductions in pain when tDCS is applied to M1 [13,15]. Previous literature has demonstrated that chronic pain results in a decreased motor threshold, increased map volume, and reduced intracortical inhibition [16]. It is believed that these changes, particularly in chronic pain patients, are caused by reduced somatosensory input, disuse of the painful limb, and the loss of muscle targets [17]. Chronic orthopedic pain inhibits M1 activity, and tDCS-induced M1 activities can lead to pain reduction [18]. Therefore, not only can tDCS decrease chronic orthopedic joint pain, but it can also amplify the pain-relieving effects of physical therapy exercises through increased M1 activity [19].

However, the current understanding of tDCS‘s efficacy on chronic orthopedic joint pain is still limited due to the differing methods used across studies. Previous systematic reviews predominantly focus on studies of single condition (e.g., chronic lower back pain, fibromyalgia, migraine headaches) without a comprehensive analysis of orthopedic-related chronic pain conditions in a single review study [20,21,22]. Since each joint operates both mechanically and neurologically in distinct ways, it is crucial to investigate the impact of tDCS on various joints throughout the body, which will help establish more systematic approaches to evaluating the clinical effectiveness of tDCS in rehabilitation. This approach will contribute to better identifying and understanding the treatment strategies for various chronic orthopedic pain conditions. Thus, the aim of our systematic review with meta-analysis is to examine the efficacy of tDCS intervention in reducing chronic orthopedic pain.

## 2. Materials and Methods

### 2.1. Protocol

The current systematic review follows the guidelines of the Preferred Reporting Items for Systematic Reviews and Meta-analyses (PRISMA) 2020 statement (See Supplementary File S1, PRISMA checklist) [23]. Only randomized controlled studies were included in this review.

### 2.2. Literature Search

We searched articles published from the inception of each database through 1 October 2022, from five electronic databases: Medline, Embase, Web of Science, CINAHL, and the Cochrane databases. Two investigators (W.A. and S.I.) conducted the literature search and study selection. The senior investigator (J.K.) then verified the search and selection process until all investigators reached a consensus on the process. We identified studies related to tDCS and chronic orthopedic pain using the following keywords: (1) “motor cortex”, “primary motor cortex”, “M1”, “dorsolateral prefrontal cortex”, “DLPFC”; (2) “transcranial”, “transcranial direct current stimulation”, “tDCS”, “direct current stimulation”; and (3) “pain”. Chronic orthopedic pain was defined as persisting or recurring pain affecting bone, joint, muscles or related soft tissue. We also used various combinations of these keywords. Additional references were sought from the articles and reviews retrieved. All search results were exported from each electronic database for subsequent screening.

### 2.3. Study Selection

We selected studies based on population, intervention, comparison, outcome, and study design (PICOS) inclusion criteria. We targeted a population comprising human subjects with chronic joint pain, excluding cases associated with acute pain, experimental pain, and neurologically related diseases. The interventions of interest were those involving tDCS applied to cerebral cortices linked with pain mechanisms. Studies using tDCS either independently or in combination with other standard therapies were included. Eligible studies compared tDCS with control, sham tDCS, or conventional therapeutic interventions for pain management. Our primary outcome was pain intensity, measured by the Visual Analogue Scale (VAS) or Numerical Rating Scale (NRS) [21].

In the current reviewer, data extraction was performed from each included study by the authors (W.A. and S.I.). We extracted data regarding sample characteristics, study design, tDCS treatment characteristics, characteristics of adjunct treatments (if applicable), and pain outcomes. We made attempts to contact the authors of studies twice via email in case they did not report necessary data for the analysis. If we received no response, we carefully considered excluding those studies from the analysis in order to systematically proceed with the review. If data such as the VAS score were only presented in graphical form, we estimated the score from the graph using a calibrated ruler. All post-treatment data used were from the first data collection following the completion of the treatment plan. Information from each study was independently extracted and compiled into a systematic spreadsheet for further analysis. We collected the mean and standard deviation values of pain intensity scores, such as VAS and NRS, before and after the tDCS intervention for pain outcomes. Pain scores reported on a 0–100 scale were converted to a 0–10 score to facilitate meta-analysis [24].

### 2.4. Assessment of Methodological Quality

The methodological quality of the studies was assessed using the PEDro scale, a tool widely utilized to evaluate the risk of bias in randomized controlled trials within systematic reviews [25,26,27]. On the PEDro scale, a higher score reflects greater internal validity and is, therefore, considered high-quality evidence. Scores of 0–4 are interpreted as ‘low quality’, scores of 5–7 are interpreted as ‘medium quality’, and scores of 8–10 are interpreted as ‘high quality’.

### 2.5. Statistical Analysis

Comprehensive Meta-Analysis software (Version 4, Biostat) was used to compute the effect sizes and perform meta-analyses to assess the efficacy of tDCS interventions on chronic orthopedic pains as measured by either the VAS or NRS scale. In instances where the standard deviation of changes of the pain outcome after the intervention was unavailable and the authors could not provide it, we followed the recommendations outlined in the Cochrane Handbook for Systematic Reviews of Interventions [28]. For example, we calculated the standard deviation of changes for each group in case they only reported the 95% confidence interval for the mean. The standard deviation of each group is obtained by dividing the width of the confidence interval by 3.92, and then multiplying by the square root of the sample size in that group [28]. A random-effects model was implemented for our meta-analysis by chronic orthopedic conditions. A subgroup analysis was conducted for studies within each pain condition which was further classified according to the number of treatment sessions: 1–5 sessions, 6–10 sessions, and >10 sessions. The effect size was estimated using Hedge’s *g*, based on pre- and post-intervention outcome measurements from both the active intervention and sham/control groups, along with pre- and post-intervention correlation data for each group. Cochran’s *Q* statistic and *I*^2^ index was utilized to evaluate between-study heterogeneity. The *I*^2^ index was interpreted as follows: >25% indicates low heterogeneity, >50% indicates moderate heterogeneity, and >75% indicates high heterogeneity. In the presence of significant heterogeneity among studies, we conducted a sensitivity analysis to examine the impact of potential biases or outliers on the overall effect size results. Due to the small number of studies recommended for such analyses (e.g., >10 studies included in the meta-analysis), we did not conduct publication bias statistics, such as the funnel plot and Egger’s test.

### 2.6. Certainty of Evidence

The certainty of the evidence was evaluated based on the algorithm applying to Grading of Recommendations, Assessment, Development, and Evaluations (GRADE) [29]. The level of evidence was downgraded in the presence of any of the following factors: low number of participants within pooled analysis, risk of bias, and heterogeneity in meta-analysis. There are four grades of evidence quality: high, moderate, low, and very low. Each area began with high evidence grades and was adjusted based on the outcomes of the aforementioned factors [29].

## 3. Results

### 3.1. Search Findings

**Figure 1:**
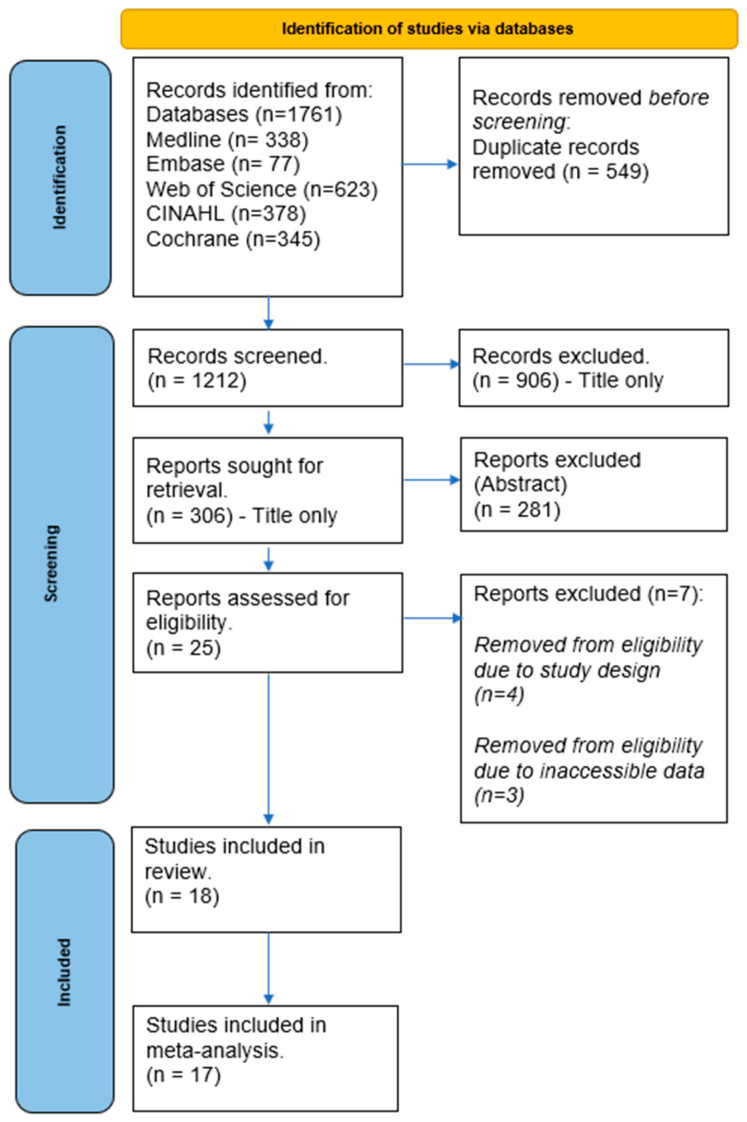
PRISMA flow chart.

Our search strategy yielded a total of 1761 studies from the electronic database searches. We removed 549 duplicates, and a further 1212 were excluded after screening titles and abstracts. A detailed review of 25 studies was conducted for eligibility using full-text screening. Four studies were further excluded due to their study design, three were excluded for not reporting the pain scale outcome measures, and one was disqualified due to a different tDCS intervention reported. Ultimately, eighteen studies met the eligibility criteria and were included in the review. A flowchart detailing the study selection process is depicted in Figure 1.

### 3.2. Study Characteristics

The study characteristics are described in Table 1. A total of 18 studies [13,14,15,19,30,31,32,33,34,35,36,37,38,39,40,41,42,43] were included in this systematic review. Eight of these studies [19,30,31,32,37,39,40,43] evaluated the effects of tDCS on knee pain, yielding a total of 496 subjects whose ages ranged from 22.9 to 73.9 years. Seven of these studies [19,30,31,32,37,39,40] were related to knee osteoarthritis, while one study [38] was related to patellofemoral pain syndrome. Seven studies [13,14,15,20,34,35,36,42] evaluated chronic lower back pain (CLBP), involving a total of 386 subjects aged between 30.0 and 63.2 years. Two studies [33,41] on myofascial shoulder pain included a total of 45 subjects aged between 47.9 and 59.6 years. Lastly, one study [38] examining temporomandibular joint disorder involved a total of 32 subjects with an average age of 24.7 years. The key characteristics are presented in Table 1.

### 3.3. Intervention

#### 3.3.1. Knee Pain

All eight studies [19,30,31,32,37,39,40,43] utilized anodal tDCS, targeting M1 [30,31,32,37,39,40], except for one study [19] administering tDCS to the primary somatosensory cortex and the dorsolateral prefrontal cortex. Four studies [30,32,37,43] utilized tDCS as the lone therapeutic intervention, while the four others [19,30,39,40] used tDCS in conjunction with other interventions, including strengthening exercises [19,39,40] and mindfulness-based meditation [31]. 

#### 3.3.2. Lower Back Pain

Out of the seven studies investigating CLBP, six studies applied [13,14,15,34,35,36,42] anodal tDCS to M1, whereas one study [36] targeted the dorsal anterior cingulate cortex. Two of the studies [35,36] used tDCS as the lone treatment, while the other five studies [13,14,15,34,42] added adjunctive therapy to tDCS such as peripheral electrical stimulation [13,15], balance training [34], cognitive behavioral therapy [14], and strengthening exercises [42]. 

#### 3.3.3. Shoulder Pain

Choi et al. [33] examined the effects of tDCS on both M1 and the dorsolateral prefrontal cortex. On the other hand, Sakrajai et al. [41] only investigated the impact of tDCS on M1 in shoulder pain. Both studies exclusively evaluated the effects of tDCS alone.

#### 3.3.4. Orofacial Pain

Oliveira et al. [38] evaluated the effects of tDCS on M1 in patients with temporomandibular joint disorder. This study compared the effects with a sham tDCS condition in conjunction with strengthening exercises [38].

#### 3.3.5. Sham and Blinding

For the knee pain condition, all studies, except for Sajadi et al. [40], used sham tDCS as the control group, whereas Sajadi et al. [40] compared the effects of tDCS to transcutaneous electrical nerve stimulation. Five studies. [19,31,37,39,43] used a 30 s ramp-up-and-down procedure, while two studies [30,32] used a 10 s ramp-up and down procedure. One study did not provide any specific information regarding the sham tDCS procedure. All of the studies [19,30,31,37,40,43] were double-blinded studies, involving participants, experimenters, or an outcome rater, except for two studies [32,39] which used a single-blind of patients. 

For the lower back pain condition, all studies utilized a sham tDCS treatment as the control group. Three studies [13,36,42] used a 30 s ramp-up-and-down procedure, while three studies [15,34,35] used a 10 s ramp-up-and-down procedure. One study [14] did not provide any specific information regarding the sham tDCS procedure. All of the studies [13,14,34,35,36,42] were double-blinded studies involving participants, experimenters, or outcome raters, except for one study [15], which used a single-blind of patients. 

For the shoulder pain, both studies used sham tDCS treatment in the control group and utilized a 30 s ramp-up-and-ramp-down procedure for the sham tDCS application. Choi et al. [33] used double-blinding while Sakrajai et al. [41] used the single-blinding of patients. 

For the orofacial pain condition, Oliveria et al. used a 30 s ramp-up-and ramp-down procedure for the sham tDCS application with the double-blinding of patients and experimenter. 

### 3.4. Quality Assessment

Two reviewers (W.A. and S.I.) independently assessed the quality of studies using the PEDro scale. Cohen’s kappa inter-rater reliability analysis demonstrated an almost perfect agreement (91.9%) between the two reviewers (K = 0.464, 95% CI = 0.24–0.69). A final consensus was achieved on all items through discussion. Table 2 presents the individual PEDro scores for all studies.

### 3.5. Meta-Analysis

We identified three categories of chronic orthopedic joint pain suitable for the meta-analysis, including knee pain (k = 8), CLBP (k = 7), and shoulder pain (k = 2). Only one study [38] evaluated chronic orthopedic joint pain on the orofacial area. Therefore, the study with orofacial pain was not included in the meta-analysis but was reviewed individually. Furthermore, the number of treatment sessions was categorized into (1) less than five treatment sessions; (2) between 6 and 10 treatment sessions; and (3) more than 10 sessions.

#### 3.5.1. Knee Pain

**Figure 2:**
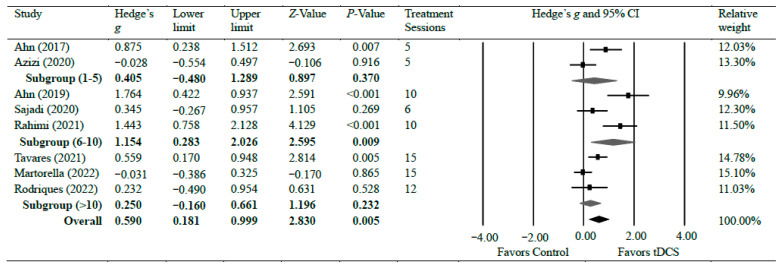
Forest plot comparing effects of tDCS to control group on knee pain.

Eight studies [19,30,31,32,37,39,40,43] investigated the efficacy of anodal tDCS on chronic knee pain. Overall, the meta-analyses found a significant reduction in chronic knee pain with moderate effects (*g* = 0.59, 95% CI = 0.18 to 0.10, *p* = 0.005). There was significant heterogeneity among studies associated with the observed effects (Tau^2^(1) = 0.26, *p* < 0.001, *I*^2^ = 77%). However, the overall effect size was consistent. We conducted a further sensitivity analysis. The analysis revealed that removing any individual study from the analysis did not significantly alter the overall effect size result, except for two studies that resulted in a slight change in the overall effect to a small effect: Ahn et al. [30] (*g* = 0.45) and Rahimi et al. [19] (*g* = 0.47), indicating that the overall effect size results of the meta-analysis are relatively consistent and not influenced by individual studies, except for the two specified studies. There was moderate certainty of evidence supporting pain reduction following tDCS. As for the subgroup analysis, two studies evaluated pain following a short treatment plan of five sessions of tDCS intervention and observed no significant difference (*g* = 0.40, 95% CI = −0.48 to 1.29, *p* = 0.370) [30,32]. Three studies [19,31,39] evaluated pain after a moderate treatment plan of 6–10 sessions of tDCS intervention and observed a strong effect of tDCS on pain reduction when compared with control groups (*g* = 1.15, 95% CI = 0.28 to 2.03, *p* = 0.009). Two studies [37,43] evaluated pain after 12 and 15 sessions of tDCS intervention and found no significant difference between the groups (*g* = 0.25, 95% CI = −0.16–0.66, *p* = 0.232). Figure 2 shows Hedge’s *g* effect sizes on knee pain after tDCS.

#### 3.5.2. Lower Back Pain

**Figure 3:**
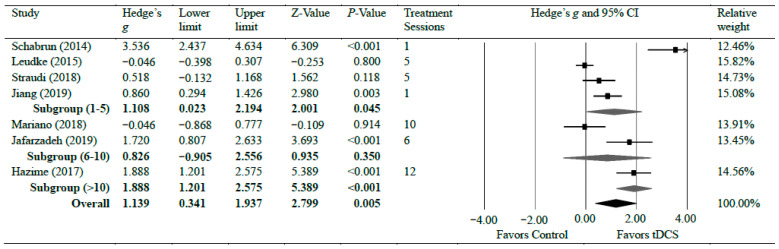
Forest plot comparing effects of tDCS to control group on lower back pain.

Seven studies [13,14,15,34,35,36,42] investigated the efficacy of anodal tDCS on CLBP. The meta-analyses found a significant reduction in CLBP with strong effects (*g* = 1.14, 95% CI = 0.34 to 1.94, *p* = 0.005). Statistical analysis found significant heterogeneity among studies associated with the observed effects (Tau^2^(1) = 1.02, *p* < 0.001, *I*^2^ = 90%). However, a further sensitivity analysis showed that excluding any individual study did not significantly change the overall effect size, except for a study by Shabrun et al. [15] which resulted in a moderate effect size (*g* = 0.79), indicating the robustness of the overall effect size following tDCS on CLBP. There was a moderate certainty of evidence supporting pain reduction following tDCS in patients with CLBP. Subgroup analyses were conducted based on the number of treatment sessions. Four studies [14,15,35,42] evaluated pain after a shorter treatment plan of tDCS, which included a single session, four sessions, and five sessions. The shorter treatment plan of tDCS had a strong effect on pain reduction with CLBP (*g* = 1.10, 95% CI = 0.02–2.19, *p* = 0.045). Two studies [34,36] evaluated pain during between 6 and 10 sessions of tDCS. No significant difference was observed in pain reduction when patients received between 6 and 10 sessions (*g* = 0.83, 95% CI = −0.91 to 2.56, *p* = 0.350). One study [13] assessed pain after 12 sessions and found a strong reduction in pain after the tDCS application (*g* = 1.88, 95% CI = 1.20–2.58, *p* < 0.001) [13]. Figure 3 shows Hedge’s *g* effect sizes on lower back pain after tDCS.

#### 3.5.3. Shoulder Pain

**Figure 4:**
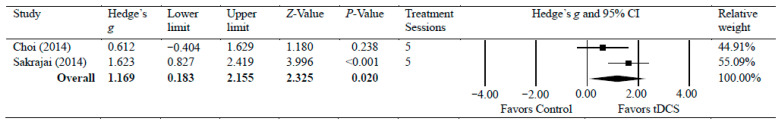
Forest plot comparing effects of tDCS to control group on shoulder pain.

Two studies [33,41] investigated the efficacy of anodal tDCS on chronic shoulder pain [33,40]. The meta-analyses found a significant reduction in chronic shoulder pain, with moderate effects (*g* = 1.17, 95% CI = 0.18 to 2.16, *p* = 0.020). Statistical analysis found no significant heterogeneity among studies associated with the observed effects (Tau^2^(1) = 0.29, *p* = 0.125, *I*^2^ = 58%). There was a low certainty of evidence supporting pain reduction following tDCS with chronic shoulder pain. Figure 4 shows Hedge’s *g* effect sizes on shoulder pain after tDCS.

#### 3.5.4. Orofacial Pain

Olivera et al. [38] investigated the efficacy of anodal tDCS on temporomandibular pain. The results of this study did not demonstrate a statistically significant difference in pain reduction between the two groups (*g* = 0.00, 95% CI = −0.68–0.68, *p* = 1.000).

## 4. Discussion

We systematically evaluated the effects of tDCS on pain levels in subjects with chronic orthopedic joint pain. Our results demonstrate that tDCS effectively reduces pain levels in subjects with chronic knee, lower back, and shoulder-joint pain. These findings are consistent with the systematic review by Cai et al. [21] which found that tDCS was effective in treating migraine headaches. However, other reviews indicated no clinical effects of tDCS on chronic pain such as CLBP [20], chronic pelvic pain [12], and fibromyalgia [22].

### 4.1. Knee Pain

We found a statistically significant reduction in chronic knee pain after tDCS. We also found that the treatment effect was modulated by the treatment length, showing a pain reduction after 5–10 sessions of tDCS intervention in patients with chronic knee pain. Our results suggest that extended tDCS showed no additional effect when there are more than 10 sessions of treatment. Upon examining the design of three studies [19,31,39] implementing 5–10 sessions of tDCS, all involved knee osteoarthritis, and the two studies [19,31,39] showing the most notable pain reductions used additional therapies with tDCS: Ahn et al. [31] added mindfulness-based meditation, and Rahimi et al. [19] utilized physical therapy. This suggests that patients with knee osteoarthritis may be more receptive to tDCS with additional therapy when the treatment plan is of moderate length [37,43]. A recent review supported the selective treatment effects of tDCS, demonstrating that the groups receiving 5–10 sessions of tDCS showed a significant improvement in chronic pain [44].

Pain intensity in knee osteoarthritis often does not correlate with the severity of structural damage, which likely indicates central sensitization as a possible explanation for the disproportionate pain intensity in this chronic pain population [33]. It has been proposed that, specifically in the cases of knee osteoarthritis, there is evidence of deviations in central pain processing, leading to a mismatch between pathophysiology and reported pain intensity [45]. Additionally, the structural changes in the case of osteoarthritis are generally irreversible, yet pain modification is still possible by modulating the pain processing areas of the brain and facilitating downstream pain inhibition [46]. Therefore, tDCS potentially offer a safe, non-pharmacological therapy that can effectively be used on its own or in conjunction with other therapy to reduce the pain intensity in patients suffering from chronic knee pain.

### 4.2. Lower Back Pain

A significant pain improvement after tDCS in patients with CLBP in the current study contrasts with the previous review by Alwardat et al. [20], which showed a non-significant effect of tDCS on pain reduction with CLBP. The most likely explanation for this difference in results is that our study evaluated the effectiveness of tDCS on pain scores directly following the last treatment session, while the analysis performed by Alwardat et al. [20] considered the pain scores after the final follow-up, which could be as long as 6 months after the last treatment. Consequently, their study addresses whether the benefits of tDCS were maintained weeks or months after treatment, whereas our review evaluated the direct impact of tDCS on pain scores upon the conclusion of the treatment plan.

In contrast to our review of the effects of tDCS on chronic knee pain, the treatment plans of 1–5 sessions and 11–15 sessions showed a statistically significant change in pain outcomes with CLBP (Figure 3). Hazime et al. [13] found that tDCS alone was not sufficient to provide long-lasting relief unless combined with peripheral electrical stimulation (PES). Schabrun et al. [15] found that, when tDCS was combined with PES, even when the duration of the treatment plan was short, there was a significant reduction in pain levels in subjects suffering from CLBP. Therefore, it may be the case that tDCS is best utilized with additional therapy for treating CLBP so that both the central and peripheral mechanisms are addressed to provide long-term relief.

Although CLBP is a non-specific diagnosis and can include various pathologies, such as disc herniations, facet syndrome, and spinal stenosis, there still appears to be a benefit to utilizing tDCS to change the pain experience of the subject. In the systematic review by Alwardat et al. [20], they suggest that the lack of significant findings may be due to the non-specific diagnosis and differences in pain mechanism among subjects. tDCS may provide temporary relief through central pain inhibition but does not address the underlying pathoanatomical dysfunction required for long-term benefit [47].

### 4.3. Shoulder Pain

We analyzed two clinical trials that evaluated the effects of tDCS on chronic shoulder pain, formally diagnosed by myofascial pain syndrome. Myofascial pain syndrome is caused by trigger points that can lead to plastic changes in the central nervous system pain pathway and ultimately central sensitization [48]. The pooled data from Choi et al. [33] and Sakrajai et al. [41] demonstrated a statistically significant difference in pain scores when compared with sham tDCS. Both studies [33,41] compared tDCS with additional therapy to sham intervention. Choi et al. [33] compared both primary M1 tDCS and DLPFC tDCS to sham and both experimental groups demonstrated a statistically significant improvement. The rationale laid out by Choi et al. [33] for the mechanism of tDCS to the DLPFC is that it has been previously demonstrated to reduce pain thresholds in healthy subjects [49]. This research may open up the opportunities for future studies involving the use of tDCS on the DLPFC under other chronic orthopedic conditions.

Sakrajai et al. [41] also evaluated the effects of tDCS on myofascial pain syndrome in addition to traditional physical therapy. Subjects who received tDCS in additional to physical therapy showed a statistically significant improvement in pain scores relative to the group that received sham tDCS. As seen in the discussion paragraphs regarding knee and CLBP, tDCS consistently appears to demonstrate an additive effect to traditional therapies for these conditions in addition to demonstrate the benefit as a standalone therapy as seen in Choi et al. [33]. While the body of literature for the effects of tDCS on shoulder pain is limited at this time, the studies included in this review show promise for future research in this joint region.

### 4.4. Other Chronic Orthopedic Joint Pain

Our review included one study [38] evaluating the effects of tDCS on orofacial pain in patients with temporomandibular joint disorders, which did not demonstrate a statistically significant difference in pain scores when comparing tDCS with sham control prior to physical therapy. Currently, there are a few studies evaluating the effects of tDCS on chronic orthopedic joint pain apart from CLBP, knee pain, and shoulder pain. More research is needed in other joint conditions.

### 4.5. Clinical Implication

Chronic orthopedic joint pain has been challenging for the medical community in terms of finding an effective treatment due to central sensitization, a phenomenon in which the patient’s pain experience is no longer tied to their injury but rather the result of re-wiring in the brain [1,50]. As chronic pain can aggravate with age, tDCS may offer potential as a non-pharmacological alternative, especially for older adults with a high risk of opioid disorders due to prolonged medication use.

Currently, tDCS is still being used, primarily for research purposes, and clinical uses have been focused on treating neurological conditions such as Alzheimer’s disease, epilepsy, depression, and tinnitus. Its clinical use into orthopedic medicine, however, has been slow due to limited high-quality federally funded clinical trials and insufficient training within hospitals [51]. However, the application of tDCS requires adjustments based on the specific joint area affected. Still, our findings suggest potential use of tDCS for addressing chronic orthopedic joint pain and encouraging further research in this area.

### 4.6. Limitations

Our study is not without limitations. The primary limitation of this study is that the majority (14/18) of the clinical trials only evaluated two conditions, CLBP and knee osteoarthritis. Therefore, more studies are needed in other joint areas (e.g., ankle, hip, etc.). We acknowledge the limited number of studies included due to our inclusion criteria of RCTs to analyze the highest available evidence for the current topic, which may pose a potential risk of selection bias. However, including other low-quality evidence may contribute to a potential problem in concluding the efficacy of tDCS on chronic orthopedic pain patients. Additionally, our approach, while assessing the overall effectiveness of a technique in chronic orthopedic pain, might not fully align with the evolving trend of personalized treatments in physiotherapy. Furthermore, most of the studies in this review investigated the application of tDCS to M1 because mechanistic studies showed that inducing the brain activity of M1 could improve central pain pathways via communication with the thalamus, brainstem, cingulate gyrus, and prefrontal cortex [52,53]. One study by Choi et al. [33] showed the comparable effects of DLPFC-tDCS on pain relief to M1-tDCS. DLPFC, which is considered a critical brain area for regulating the perception of pain, needs to be explored further. Lastly, we categorized (subgrouped) our included studies based on the total number of treatment sessions. Future review studies may consider other stratifications such as by stimulation site or supplementary treatments applied during the tDCS treatment.

## 5. Conclusions

The findings suggest that tDCS can be considered either as a stand-alone treatment option or as a supplemental treatment for those suffering from chronic orthopedic joint pain, indicating its potential efficacy in managing chronic pain. However, the effectiveness of the tDCS treatment varies depending on the specific pain region. Our findings suggest the importance of implementing individualized treatment plans when considering tDCS for chronic pain conditions. Future research is warranted to determine the optimal treatment duration for different types of chronic orthopedic joint pain.

## Figures and Tables

**Table 1:** Characteristics of included studies.

Pain Area	Author (Year)	Total (tx/c) ^a^	Age (Mean)	Pain Condition	tDCS Mode	tDCS Placement	Control	Intensity (mA)	Duration (min)	Total Sessions (S) Frequency (F) Length of Tx (L)	Other Interventions	Pain Outcome	Finding ^e^
Knee	Ahn (2017) [30]	40 (20/20)	60.0	Knee OA	Anodal	M1 (Contra) ^c^	Sham	2	20	S:5; F:Daily; L:1 wk	None	NRS	Improved
	Ahn (2019) [31]	30 (15/15)	59.5	Knee OA	Anodal	M1 (Contra) ^c^	Sham	2	20	S:10; F:Daily; L:2 wk	MBM	NRS	Improved
	Azizi (2021) [32]	54 (27/27)	58.9	Knee OA	Anodal	M1 (Contra) ^c^	Sham	2	20	S:5; F:Daily; L:1 wk	None	VAS	Improved
	Sajadi (2020) [40]	40 (20/20)	58.1	Knee OA	Anodal	M1 (Contra) ^c^	PES	2	20	S:6; F:3 x/wk; L:2 wk	EX	VAS	NC
	Rahimi (2021) [19]	80 (20/20)	58.8	Knee OA	Anodal	M1, S1, DLPFC (Contra) ^c^	Sham	1	20	S:10; F:Daily; L:2 wk	EX	VAS	Improved
	Tavares (2021) [43]	104 (51/53)	73.9	Knee OA	Anodal	M1 (Contra) ^c^	Sham	2	20	S:15; F:Daily; L:3 wk	None	VAS	Improved
	Martorella (2022) [37]	120 (60/60)	66.0	Knee OA	Anodal	M1 (Contra) ^c^	Sham	2	20	S:15; F:Daily; L:3 wk	None	NRS	Improved
	Rodrigues (2022) [39]	28 (14/14)	22.9	PFPS	Anodal	M1 (B/L) ^c^	Sham	2	20	S:12; F:2–3/wk; L:6 wk	EX	VAS	Improved
Lower back	Schabrun (2014) [15]	16 (16) ^b^	30.0	CLBP	Anodal	M1 (Contra) ^c^	Sham	2	30	S:1	PES	VAS	NC
	Leudke (2015) [14]	135 (67/68)	44.5	CLBP	Anodal	M1 (Left) ^c^	Sham	2	20	S:5; F:Daily; L:1 wk	CBT	VAS	NC
	Hazime (2017) [13]	92 (23/23) ^b^	52.6	CLBP	Anodal	M1 (Contra) ^c^	Sham	2	20	S:12; F:3x/wk; L:4 wk	PES	VAS	NC
	Mariano (2019) [36]	21 (10/11)	63.2	CLBP	Anodal	dACC (Left) ^d^	Sham	2	20	S:10; F:Daily; L:2 wk	None	VAS	NC
	Straudi (2018) [42]	35 (18/17)	55.1	CLBP	Anodal	M1 (Contra or B/L) ^c^	Sham	2	20	S:5; F:Daily; L:1 wk	EX	VAS	Improved
	Jafarzadeh (2019) [34]	36 (12/12) ^b^	31.6	CLBP	Anodal	M1 (Left) ^c^	Sham	2	20	S:6; F:3x/wk; L:2 wk	Postural training	VAS	Improved
	Jiang (2020) [35]	51 (26/25)	42.0	CLBP	Anodal	M1 (Dom) ^c^	Sham	2	20	S:1	None	NRS	Improved
Shoulder	Choi (2014) [33]	14 (8/6)	59.6	MPS	Anodal	M1, DLPFC (Contra) ^c^	Sham	2	20	S:5; F:Daily; L:1 wk	None	VAS	Improved
	Sakrajai (2014) [41]	31 (16/15)	47.9	MPS	Anodal	M1 (Contra) ^c^	Sham	2	20	S:5; F:Daily; L:1 wk	None	VAS	Improved
Head	Oliviera (2015) [38]	32 (16/16)	24.7	TMJD	Anodal	M1 (Contra) ^c^	Sham	2	20	S:5; F:Daily; L:1 wk	EX	VAS	NC

^a^ indicates the number of subjects in the tDCS treatment group and control/sham group. ^b^ If the sum of subjects in the two groups does not match the total number of subjects, it can indicate a randomized crossover trial or a study with more than two groups. ^c^ studies with a reference electrode were placed on Fp1/Fp2. ^d^ indicates a study with a reference electrode placed on the contralateral mastoid. **^e^** the statistical results of each individual study were used to determine the findings on pain reduction after the active tDCS intervention versus comparison groups. Abbreviations: NC, no change; Tx: treatment; OA: osteoarthritis; CLBP: chronic lower back pain; MPS: myofascial pain syndrome; TMJD: temporomandibular joint disorder; PFPS: patellofemoral pain syndrome; NRS: numeric rating pain scale; VAS: visual analogue scale; M1: motor cortex; DLPFC: dorsolateral prefrontal cortex; S1: somatosensory cortex; dACC: dorsal anterior cingulate cortex; MBM: mindfulness-based meditation; PES: peripheral electrical stimulation; EX: strengthening exercises; Contra: contralateral to painful side; B/L: bilateral application; Dom: dominant hemisphere.

**Table 2:** Quality assessment (PEDro scale).

Pain Area	Author (Year)	Score	Random Allocation	Concealed Allocation	Group Similar at Baseline	Subject Blinding	Therapist Blinding	Assessor Blinding	<15% of Dropouts	Intention-to-Treat Analysis	Between-Group Comparison	Point Estimates and Variability
Knee	Ahn (2017) [30]	9	O	O	O	O	O	O	-	O	O	O
	Ahn (2019) [31]	8	O	O	O	O	O	-	-	O	O	O
	Sajadi (2020) [40]	10	O	O	O	O	O	O	O	O	O	O
	Azizi (2021) [32]	8	O	O	O	O	O	-	-	O	O	O
	Rahimi (2021) [19]	9	O	O	O	O	O	O	-	O	O	O
	Tavares (2021) [43]	10	O	O	O	O	O	O	O	O	O	O
	Martorella (2022) [37]	10	O	O	O	O	O	O	O	O	O	O
	Rodrigues (2022) [39]	8	O	O	O	O	O	-	-	O	O	O
	Average	9	100%	100%	100%	100%	100%	63%	38%	100%	100%	100%
Lower back	Schabrun (2014) [15]	10	O	O	O	O	O	-	-	O	O	O
	Leudke (2015) [14]	9	O	O	O	O	O	-	O	O	O	O
	Hazime (2017) [13]	10	O	O	O	O	O	O	O	O	O	O
	Mariano (2019) [36]	8	O	O	O	O	O	O	O	O	O	O
	Straudi (2018) [42]	10	O	O	O	O	O	O	O	O	O	O
	Jafarzadeh (2019) [34]	10	O	O	O	O	O	O	O	O	O	O
	Jiang (2020) [35]	10	O	O	O	O	O	-	O	O	O	O
	Average	9.6	100%	100%	100%	100%	100%	63%	87%	100%	100%	100%
Shoulder	Choi (2009) [33]	10	O	O	O	O	O	O	O	O	O	O
	Sakrajai (2014) [41]	10	O	O	O	O	O	O	O	O	O	O
	Average	10	100%	100%	100%	100%	100%	100%	100%	100%	100%	100%
Head	Oliviera (2015) [38]	10	O	O	O	O	O	O	O	O	O	O
	Total Avg	10	100%	100%	100%	100%	100%	72.2%	66.7%	100%	100%	100%

“O” indicates satisfactory; “–” indicates not satisfactory.

## Data Availability

Data are available from the corresponding author upon request. The data are not publicly available due to privacy and ethical restrictions.

## Supplementary Materials

The following supporting information can be downloaded at: https://www.mdpi.com/article/10.3390/brainsci14010066/s1, Table S1: PRISMA checklist.
